# Endoscopic septotomy for a rare colorectal anastomotic complication after elective laparoscopic sigmoidectomy for diverticular disease

**DOI:** 10.1055/a-2598-3990

**Published:** 2025-05-28

**Authors:** Mario Gagliardi, Carmela Abbatiello, Mariano Sica, Carlo Soldaini, Michele Fusco, Attilio Maurano, Claudio Zulli

**Affiliations:** 1 18596 Digestive Endoscopy Unit, University Hospital ‘San Giovanni di Dio e Ruggi dʼAragona’ ‘Gaetano Fucito’ Location, Mercato San Severino, Italy; 2 18678 Endoscopy Unit, University Hospital ‘San Giovanni di Dio e Ruggi dʼAragona’, Salerno, Italy

A 55-year-old woman who complained of colicky pain, constipation, and abdominal fullness was referred to our endoscopy center by the surgical unit. The patient had undergone elective sigmoidectomy for diverticular disease 5 weeks previously and had received 1 week of polyethylene glycol (PEG)-based laxatives and antispasmodic therapy without clinical improvement.

As a colorectal anastomotic stricture was suspected, rectoscopy was scheduled and performed, showing a thick septum that was partially occluding the colorectal anastomosis, which appeared to be freely passable with a gastroscope ( Fig. 1 **a** ). During the same procedure, an endoscopic septotomy was performed using a single-use Triangle Tip electrosurgical knife ( Fig. 1 **b** ). A minimal amount of intraprocedural bleeding occurred and was treated by the application of through-the-scope clips ( Fig. 2 ). No other complications were observed ( Video 1 ).

**Fig. 1:**
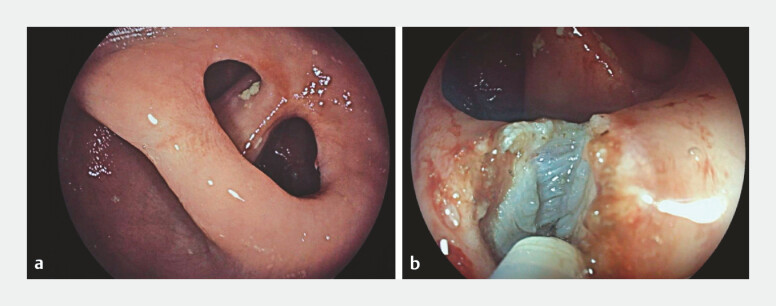
Rectoscopic views showing: **a** a thick septum that was partially occluding the colorectal anastomosis; **b** endoscopic septotomy being performed with a single-use Triangle Tip electrosurgical knife.

**Fig. 2:**
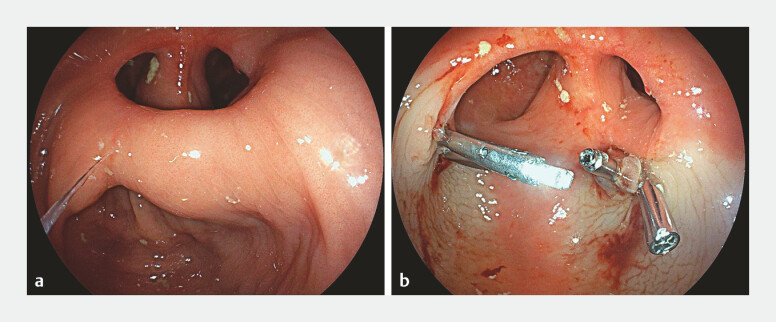
The efficacy of endoscopic septotomy is shown in comparable images: **a** pre-procedure; **b** 4 weeks after the treatment.

Endoscopic septotomy is performed for a rare colorectal anastomotic complication after elective laparoscopic sigmoidectomy for diverticular disease.Video 1

The patient returned 4 weeks later to undergo a follow-up rectoscopy, which showed the septotomy scarring and a wide anastomotic lumen. No bleeding, tenesmus, or constipation were reported by the patient over the next weeks.

Anastomotic stricture/stenosis is a common complication after colorectal anastomosis 1 but, to the best of our knowledge, we here report for the first time endoscopic septotomy for the treatment of a colorectal anastomotic septum. This treatment may become a valid alternative to revisional surgery for this rare colorectal anastomotic complication after elective laparoscopic sigmoidectomy.

Endoscopy_UCTN_Code_TTT_1AQ_2AF

## Notes

Endoscopy E-Videos https://eref.thieme.de/e-videos
*E-Videos* is an open access online section of the journal *Endoscopy* , reporting on interesting cases and new techniques in gastroenterological endoscopy. All papers include a high-quality video and are published with a Creative Commons CC-BY license. Endoscopy E-Videos qualify for HINARI discounts and waivers and eligibility is automatically checked during the submission process. We grant 100% waivers to articles whose corresponding authors are based in Group A countries and 50% waivers to those who are based in Group B countries as classified by Research4Life (see: https://www.research4life.org/access/eligibility/ ). This section has its own submission website at https://mc.manuscriptcentral.com/e-videos .
